# Occlusal disharmony attenuates inhibitory synaptic transmission in the medial prefrontal cortex and contributes to anxiety

**DOI:** 10.1016/j.bj.2025.100909

**Published:** 2025-08-21

**Authors:** Juan Li, Jiayao Zhang, Ming Xu, Qi Zhang, Weicai Liu

**Affiliations:** a Shanghai Engineering Research Center of Tooth Restoration and Regeneration & Tongji Research Institute of Stomatology & Department of Prosthodontics, Shanghai Tongji Stomatological Hospital and Dental School, Tongji University, Shanghai, China; b Shanghai Key Laboratory of Brain Functional Genomics (Ministry of Education), Affiliated Mental Health Center (ECNU), School of Psychology and Cognitive Science, East China Normal University, Shanghai, China; c Shanghai Changning Mental Health Center, Shanghai, China

**Keywords:** Occlusal disharmony, Anxiety, The medial prefrontal cortex, Mifepristone, Electrophysiological, GABA

## Abstract

**Background:**

It has been well-validated that Occlusal Disharmony (OD) induces negative emotions, especially anxiety. While it leads to considerable frustration for both doctors and patients, its underlying mechanisms remain unclear, and effective treatment has been lacking. The present study aims to explore the pathological mechanisms of OD induced anxiety and to find a straightforward yet effective treatment strategy for this ostensibly intricate clinical phenomenon.

**Material and methods:**

OD mice were established through binding a metal tube to their right mandibular incisor. Then, the mental state of the mice was assessed by various behavioral experiments. Additionally, the mood of OD mice was also evaluated similarly before and after the injection of mifepristone. Furthermore, neuronal excitability in the mPFC was examined by immunofluorescence and electrophysiology, with the potential mechanisms investigated through Western blotting.

**Results:**

OD mice exhibited anxiety-like behavior, and the administration of mifepristone, an anxiolytic drug, could alleviate it. Subsequently, an increase in neuronal excitability was observed, accompanied by a reduction in the frequency and amplitude of miniature inhibitory postsynaptic currents (mIPSCs) in the medial prefrontal cortex (mPFC). In addition, the expression of erb-b2 receptor tyrosine kinase 4 (ERBB4) and neuregulin 1 (NRG1), proteins associated with inhibitory neurotransmitter release, were found to be significantly downregulated in the mPFC.

**Conclusion:**

OD attenuates GABAergic synaptic transmission in the mPFC, which may be the neural mechanism of anxiety caused by OD. But this phenomenon could be effectively alleviated by anti-anxiety treatment.

## Highlights

• OD leads to anxiety in mice.
• Injecting Mifepristone could alleviate OD induced anxiety.
• OD induces a diminished inhibitory synaptic transmission and results in E/I imbalance in mPFC.
• OD reduced the GABA release in the mPFC.

## Graphical abstract

**Figure:**
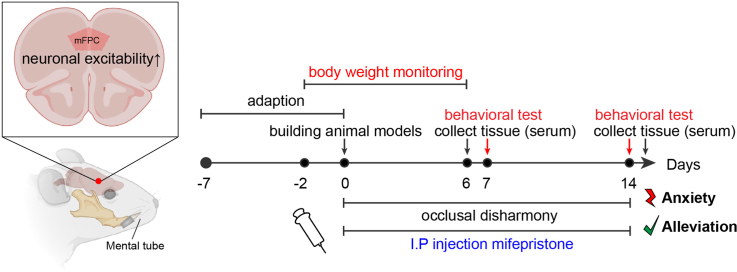

## 1 Introduction

Clinically, Occlusal Disharmony (OD) delineates a condition where interactions between opposing occlusal surfaces deviate from harmony with other tooth contacts and the anatomic and physiologic facets of the craniomandibular complex [1]. It poses a great threat to oral health in various aspects, such as periodontal tissues [2], temporomandibular joints [3], and facial muscles [4]. In recent years, a growing body of research has found that occlusion could act as a highly sensitive receptor in the central nervous system, and OD may cause patients to present with somatization symptoms and suffer from maladaptive moods (e.g., anxiety and depression) [5,6]. A survey of Japanese found that malocclusion may contribute to psychological stress in young people [7]. In animal studies, OD could also be observed to induce anxiety in rats, accompanied by elevated expression of 5-HT and 5-HT2A, a key neurotransmitter and receptor involved in regulating mood in specific brain regions [1,8]. Additionally, there is an increase in serum corticosterone (CORT), which is attributed to the activation of the hypothalamic-pituitary-adrenal (HPA) axis [9]. However, how does OD induce anxiety?

The cerebral cortex, subcortical brain regions, and limbic system together are involved in the regulation of emotions [10], and the medial prefrontal cortex (mPFC) is one of the most critical brain regions. It serves as a control center, receiving and integrating almost all neuronal output from the sensory and motor systems in the cortex and subcortical areas [11]. Almost any mPFC dysfunction can lead to neurological and psychiatric abnormalities, such as altered neuronal activity, connectivity, excitatory-inhibitory (E/I) balance, and neuronal numbers [12]. In addition, somatization effects are also present in the cortex, and it has been reported that the deactivation of the prefrontal cortex in OD patients is significantly associated with somatization [13]. This suggests that OD may act as a somatic change that interferes with the normal physiological activity of the mPFC. However, it remains unknown whether the mPFC is also involved in OD-induced anxiety and what neuromodulatory mechanisms are involved.

In this study, we showed that OD activates the HPA axis and induces anxiety-like behavior in mice, and it could be alleviated by mifepristone. In addition, inhibitory synaptic transmission in vertebral neurons was attenuated in the mPFC of OD mice, resulting in an overall increase in excitability. Further studies showed that this was due to decreased GABA release, while GABA synthesis was not affected. Taken together, these results suggest that the mPFC plays an important role in OD-induced anxiety and that there is a close relationship between oral behavior and neural activity and emotion. In addition, it also suggests that the clinical manifestations of oral diseases are not only limited to the oral cavity. These findings enrich the relationship between oral diseases and systemic diseases and provide clinicians with a more global and comprehensive diagnostic and therapeutic thinking.

## 2 Materials and methods

### 2.1 Mice

C57BL/6 male mice (7–8 weeks old), purchased from the Shanghai Reagan Biotechnology Co., Ltd., were bred in a controlled environment maintained at 23 °C, with 50 % humidity and a 12/12-h light/dark cycle. Each cage housed four to five mice, providing them unlimited access to water and food. All experimental procedures were conducted following approval of the Experimental Animal Care and Use Committee of Tongji University, in accordance with the National Institutes of Health guidelines (No: [2019]-DW-041).

### 2.2 OD model

**Fig. 1:**
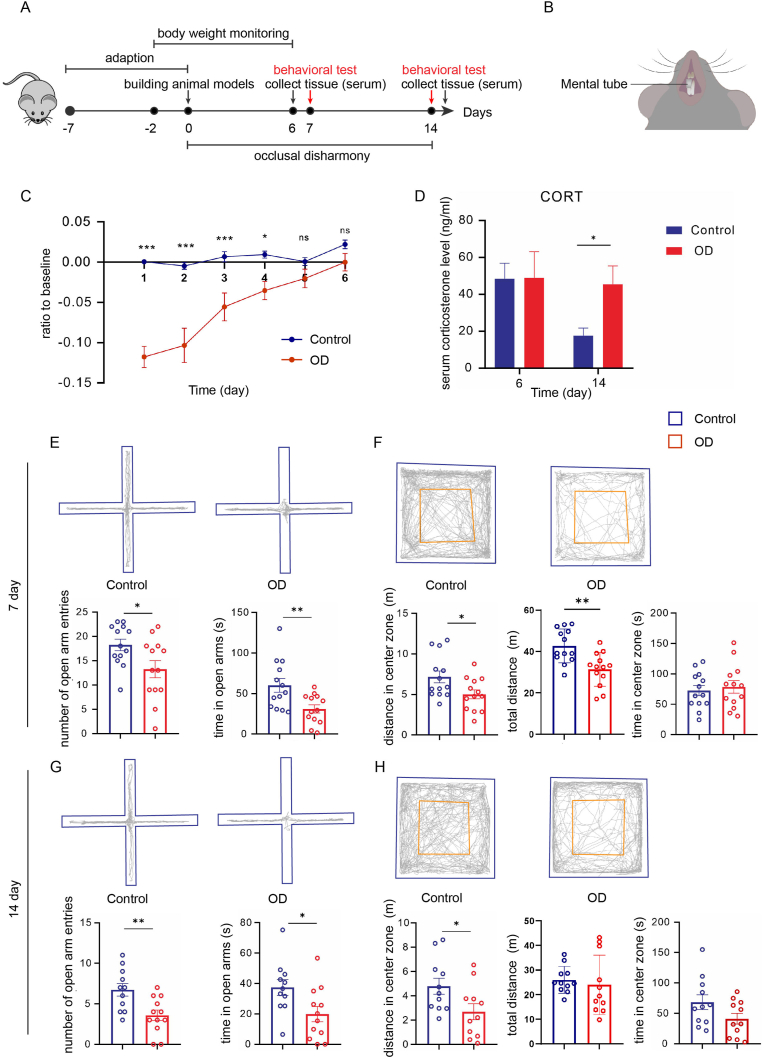
Effect of OD on body weight, serum CORT, behavioral performance. (A) The schematic timeline of the experiment. (B) Schematic diagram of OD modeling. (C) Body weight gain curve (n = 13). (D) Serum CORT concentrations (n = 6). EPM test on day 7 (E) and 14(G). OFT on day 7 (F) and 14(H) (n = 13). All data are presented as mean ± SEM; ∗, *p* < 0.05, ∗∗, *p* < 0.01, ∗∗∗, *p* < 0.001, ns, *p* > 0.05, by unpaired Student's t-test.

The OD methods were based on previous investigations and modified [14]. In brief, to induce OD, mice were given isoflurane to achieve deep anesthesia, and a metal tube was bonded onto the right mandibular incisor of the mice. The tube (2.5 mm in length) was made from syringe needles (thickness = 0.30 mm, inner diameter = 0.61 mm); one end of the metal tube was flattened by 0.5 mm. The tubes were securely attached using zinc phosphate cement [Fig. 1B]. The control group underwent an identical procedure but without the tube attachment. The mice were provided with soft-soaked and regular food, and their body weights were measured daily for one week to calculate body weight gain. $\text{Body}\;\text{weight}\;\text{gain}=\frac{\text{body}\;\text{weight}\;\text{at}\;\text{time}\;\text{point}\;t-\text{body}\;\text{weight}\;\text{before}\;\text{modeling}}{\text{body}\;\text{weight}\;\text{before}\;\text{modeling}}$

### 2.3 Behavioral procedures

Behavioral tests were conducted 7 and 14 days after modeling. Each behavioral test was performed through the animals' light cycle, videotaped by a high-resolution camera, and analyzed with Any-maze software (setting). Before testing, mice were moved into the testing chamber and given 2 h to acclimatize. The testing sequence was random.

### 2.4 Open file test (OFT)

A gray polyvinyl chloride box (40 cm × 40 cm × 30 cm) with a 20 × 20 cm central zone makes up the open field equipment. Each mouse was initially positioned at the center of the arena, after which it was permitted to roam freely for 15 min under dim light. A high-resolution camera recorded locomotor activity. In the central zones, the distance and the duration were calculated and analyzed. Between experiments, 75 % alcohol was used to clean the equipment.

### 2.5 Elevated plus maze (EPM)

The equipment was 50 cm above the ground and comprised a total of four arms—two closed and two open, each measuring 35 cm × 5 cm—along with gray roofless walls (15 cm). They were joined at a central square platform (5 × 5 cm). The experimental mouse was positioned in the central area of the equipment, with its orientation toward an open arm. Mice were free to roam around the maze for 5 min and recorded by a high-resolution camera. The number of entrances into open arms and the duration spent there were analyzed. Between experiments, 75 % alcohol was used to clean the equipment.

### 2.6 CORT assay

CORT levels were quantified in mouse serum. On the 6th and 14th days after modeling, mouse eyeballs were enucleated to collect venous blood in EP tubes. At each time point, blood samples were centrifuged for 15 min at 1000 g and 4 °C after being left to clot at room temperature for 2 h. After extraction, the serum was kept at −80 °C. In accordance with assay instructions, CORT was measured using an enzyme-linked immunosorbent assay (CUSBIO, CSB-E07969 m). All models were performed in triplicate and were within the standard curve.

### 2.7 Immunohistochemistry and imaging

Animals were given sodium pentobarbital (1 %, 100 mg/kg, intraperitoneally (i.p.)) to induce anesthesia 2 h after the OD model was constructed. They were then transcardially perfused with ice-cold 0.9 % saline, subsequently with 4 % paraformaldehyde after OD modeling for 2 h. Following dissection and overnight post-fixation at 4 °C, the brains were placed in 30 % sucrose at 4 °C for dehydration until they sank to the bottom of the 50 mL tube. a Leica CM1860 freezing microtome was employed to cut coronal brain slices to a thickness of 30 μm. Brain slices were subjected to permeabilization and blocking in PBS containing 1 % BSA, 0.3 % Triton X-100, and 10 % donkey serum for 2 h at room temperature after rinsing in PBS three times. After overnight incubation with c-FOS antibody (Synaptic Systems, 226017,1:5000) and CaMKII antibody (Abcam, ab52476, 1:500) at 4 °C, slices underwent PBS washes at room temperature for three repetitions. They were then incubated for 2 h at room temperature with a 1:1000 dilution of Alexa Fluor 488 donkey anti-rabbit IgG (Invitrogen, SA5-10038,1:1000), followed by three rinses in PBS for 10 min at room temperature. Finally, after sealing with a mounting medium containing DAPI (Southernbiotech, 0100-01), images were acquired using an Olympus vs200 microscope.

### 2.8 Drugs

Mifepristone (M8046, sigma) was solubilized in absolute ethanol for the preparation of a 50 mg/ml solution, which was then diluted with 0.9 % saline (containing 0.2 % Tween 20) immediately before injection, administered continuously for 14 days after modeling (i.p., 25 mg/kg). Saline injections were 0.9 % saline containing 0.2 % Tween 20, and the injection volume was 10 μl/g body mass by i.p.

### 2.9 Brain slice preparation

After 7–14 days of OD, whole-cell patch-clamp recordings were conducted. Mice were anesthetized with isoflurane, and coronal brain slices, each 300 μm thick, containing the mPFC were sectioned in chilled cutting buffer (26.0 mM NaHCO3, 1.25 mM NaH2PO4, 7.0 mM MgCl2, 3.0 mM KCl, 10.0 mM glucose, 212.0 mM sucrose, gassed with 5 % CO2 and 95 % O2) with a VT1000s vibratome (Leica) with reference to the mouse brain atlas. Artificial cerebrospinal fluid (ACSF) consisting of (in mM): 26 NaHCO3, 1.25 NaH2PO4, 125 NaCl, 2 CaCl2, 2.5 KCl, 1.3 MgSO4, and 20 d-glucose was used to incubate the slices. They were equilibrated with 5 % CO2 and 95 % O2, kept for 60 min to recover at 32 °C, then moved to room temperature for electrophysiological recordings. Throughout the recording and analysis process, the group identity was masked from the recorders.

### 2.10 Whole-cell patch clamp recordings

An upright microscope (Olympus) was used to visualize neurons in the slices. In the recording room, the slices were perfused with ACSF at a constant rate of around 4 mL/min and gassed with 95 % O2 and 5 % CO2. The borosilicate glass pipettes (Sutter, BF150-86-10) with an outer diameter of 1.5 mm were pulled using a Sutter Instrument P-97 micropipette puller to create patch pipettes (3–5 MΩ). Signals were acquired via a MultiClamp 700B amplifier (Axon Instruments, Foster City, CA), then transmitted via a Digidata-1550 interface (Axon Instruments) to a computer for analysis with pClamp 10.5 software. Series resistance was continually checked, and the experiment was promptly halted if the resistance fluctuated by more than 20 % throughout the recording.

For current-clamp recordings, recording electrodes made of borosilicate glass were loaded with an internal solution based on K+, containing (in mM): 130 K-gluconate, 2.5 MgCl2, 10 HEPES, 5 KCl, 4 Na2ATP, 0.6 EGTA, 0.4 Na3GTP, 10 Na phosphocreatine. Using KOH, the pH of the solution was modified to 7.2 and the osmolarity of the solution was set to 285–290 mOsm·kg-1. To assess the impact of OD on the excitability of pyramidal neurons in the mPFC, sequential currents ranging from 0 to 200 pA were injected in 20-pA steps for 400 ms. The threshold and frequency of action potentials (AP) were documented. The voltage clamp mode was employed to maintain the voltage of neurons at − 70 mV to record miniature excitatory/inhibitory post-synaptic currents (mEPSC/mIPSC). For mEPSC recordings, a K + -gluconate-based intracellular solution and ACSF containing 100 μM Picrotoxin and one μM tetrodotoxin were employed. For mIPSC recordings, a high-Cl intracellular solution and ACSF containing one μM tetrodotoxin, 50 μM D-AP5, and 10 μM NBQX were employed. The duration of the mEPSC/mIPSC recording was at least 5 min.

### 2.11 Western blotting

After 14 days of OD, mice underwent deep anesthesia and were given ice-cold 0.9 % saline perfusion. Their brains were rapidly extracted and transferred to ice, then dissected to isolate the mPFC. For total protein extraction, tissues from mPFC were homogenized in radioimmunoprecipitation assay lysis buffer for 2 min with a tissue homogenizer (Servicebio, KZ-II), followed by incubation on ice for 30 min, after which proteins underwent separation by centrifugation at 14000 rpm at 4 °C for 15 min. The Pierce bicinchoninic acid (BCA) kit (Thermo Scientific, Rockford, IL) measured the protein concentration in the supernatant. The protein concentration of the supernatant was quantified with a BCA protein assay kit (Thermo Scientific). Protein samples (18–25 μg) were mixed with protein loading buffer, boiled, and electrophoresed on a 12 %–15 % SDS-PAGE gel (CWBIO, China). Subsequently, the proteins were moved onto polyvinylidene fluoride (PVDF) membranes. At room temperature, blocking of the PVDF membranes with 5 % skim milk was performed for 2 h in TBST (150 mM NaCl, 20 mM Tris–HCl, 0.05 % Tween-20, and pH 7.5). The PVDF membranes were incubated with primary antibodies against mouse anti-GAD67 (Millipore, MAB5406, 1:1000), Rabbit anti-actin (SHAREBIO, SB-AB0035, 1:3000), rabbit *anti*-ErbB4 (Cell signaling, 4795, 1:1000) and rabbit *anti*-Neuregulin-1 (Santa Cruz Biotechnology, sc-348, 1:500) and rabbit *anti*-GAPDH (SHAREBIO, SB-AB0037, 1:3000) at 4 °C overnight. Following a TBST rinse, the PVDF membranes were incubated for 1 h and 45 min at room temperature with the HRP-conjugated goat anti-rabbit IgG secondary antibody (Proteintech, SA00001-2, 1:3000). Following a TBST rinse, the PVDF membranes were incubated with enhanced chemiluminescence solution and then processed for autoradiography. Band intensities were analyzed with ImageJ (NIH).

### 2.12 Statistical analysis

All data were analyzed and presented as the mean ± SEM. Group differences in body weight gain were determined through multiple t-tests. For various groups, we used a one-way analysis of variance (ANOVA) and subsequently performed Dunnett's post hoc multiple comparison tests. Differences between the two groups in the electrophysiological experiments and other comparisons were assessed with an unpaired Student's t-test. GraphPad Prism software (Version 8.0) was applied to perform all statistical analyses, and statistical significance was set at a P value less than 0.05.

## 3 Results

### 3.1 OD induces anxiety-like behaviors and physiological changes

The experiment was conducted in accordance with the process depicted in [Fig. 1A]. Firstly, the body weights of the mice were monitored every day to assess the feeding ability of the OD mice. The results revealed a significant short-term decrease in body weight gain in the OD group compared to the control group, returning to normal levels by the fifth day, with no statistically significant difference after that [Fig. 1C]. Then, the mental state of mice was assessed by serum hormone tests and behavioral tests for the 1 and 2 weeks after OD. It was shown that serum CORT of OD mice increased significantly on day 14, although no significant difference was observed on day 6 [Fig. 1D]. However, on both day 7 and day 14, OD mice had fewer open arms entries and spent less time in the open arms in the EPM test [Fig. 1 E&G]. Concurrently, they also had considerably lower distances in the central area of the open field [Fig. 1 F&H]. Taken together, these results all strongly suggest that occlusal disharmony contributes to an elevation in stress levels in mice.

### 3.2 Administration of mifepristone could alleviate OD-induced anxiety

**Fig. 2:**
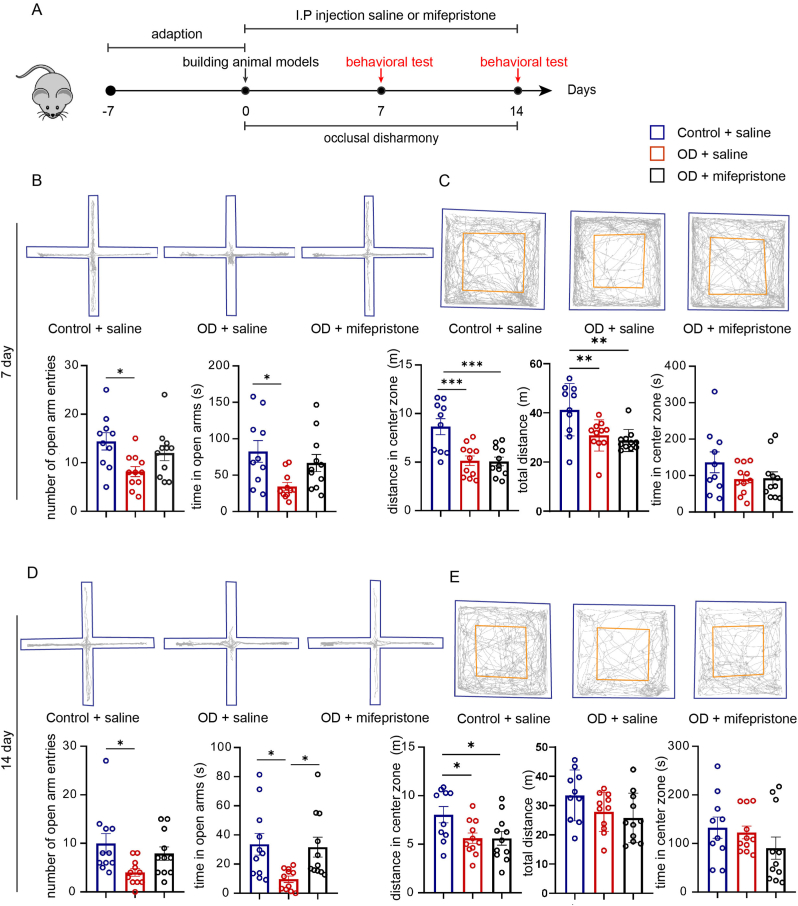
Mifepristone rescued anxiety-related behaviors in OD mice. (A) Schematic of mifepristone treatment experiment in OD mice. Number of open arm entries and time in open arms of EPM on day 7(B) and day 14(D). Time and distance in center zone of OFT on day 7(C) and day 14(E) (n = 10–11). All data are presented as mean ± SEM; ∗, *p* < 0.05, ∗∗, *p* < 0.01, ∗∗∗, *p* < 0.001, by one-way ANOVA with Dunnett's post hoc tests.

Next, mifepristone, a glucocorticoid receptor antagonist that inhibits the binding of CORT to glucocorticoid receptors, was used to explore the role of HPA axis activation with altered CORT in OD-induced anxiety. It was injected into OD mice at 25 mg/kg per day for 14 days. Then, behavioral tests were carried out on the 7th and 14th day after modeling [Fig. 2A]. As before, OD saline mice had fewer open arms entries and spent less time in the open arms than control saline mice in the EPM, but there was no difference between the OD mifepristone and saline control groups [Fig. 2 B&D]. Although a similar trend was not seen in the OFT [Fig. 2 C&E], these results still suggested that mifepristone could relieve anxiety in OD mice.

### 3.3 OD activates the mPFC and increases the excitability of neurons in the mPFC

**Fig. 3:**
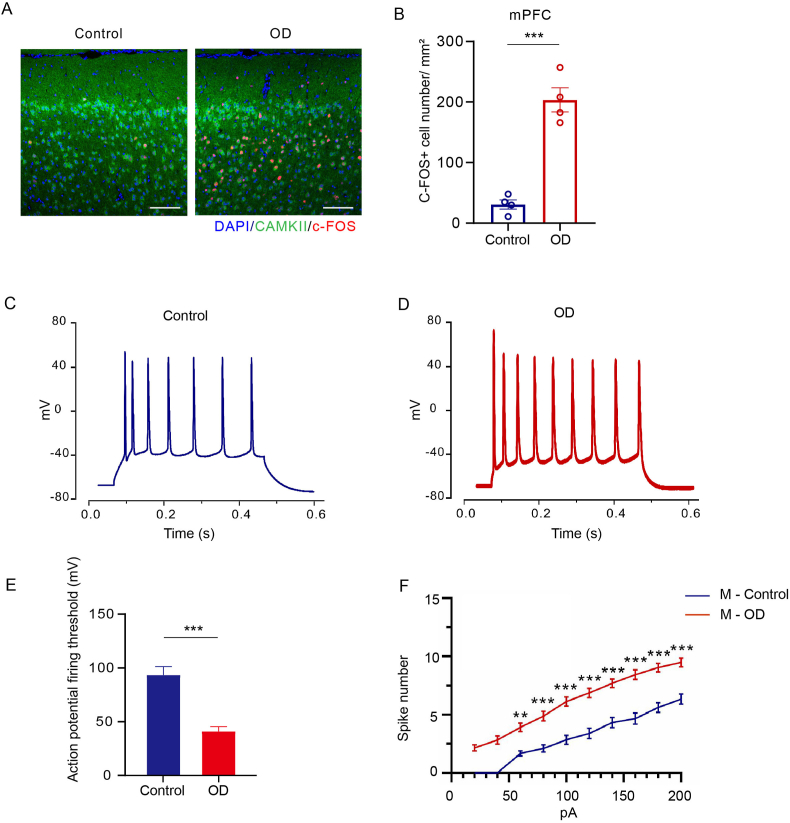
OD activated the mPFC and increased the neuronal excitability in the mPFC. (A) Representative IF imaged for c-FOS and CamkII expression in mPFC (Scale bar, 100 μm). (B) The density of c-FOS + cells in the mPFC (n = 4). (C–D) Representative traces of neuronal firing through Current-clamp recordings. (E) Action potential firing threshold (n = 20 from 4 to 6 mice per group). (F) The number of APs in a train induced by the injection of step currents and the spike number (n = 20 from 4 to 6 mice per group). All data are presented as mean ± SEM; ∗, *p* < 0.05, ∗∗, *p* < 0.01, ∗∗∗, *p* < 0.001, by unpaired Student's t-test.

As c-FOS protein detection is the most widely used method for mapping neuronal activation and studying neurocircuitry [[15], [16], [17]], immunofluorescence staining with quantification of c-FOS was then performed. The result showed that c-FOS and CaMKII expression was significantly increased in the mPFC of OD mice compared to control mice [Fig. 3 A&B]. Besides, the frequency of action potentials in response to depolarizing current steps from mPFC pyramidal neurons was measured using the patch clamp technique. The number of action potentials elicited (induced spikes) over a 400 ms interval was calculated, as the current was varied in steps of 20 pA from 0 pA to 200 pA. Depolarizing currents evoked higher firing in the OD mice than in the control mice. Similarly, less current was required to drive the cell to fire spikes at a given frequency [Fig. 3C–F]. To conclude, these results all indicated that OD activates the mPFC and leads to an increase in neuronal excitability.

### 3.4 OD impairs the inhibitory synaptic function of pyramidal neurons in the mPFC

**Fig. 4:**
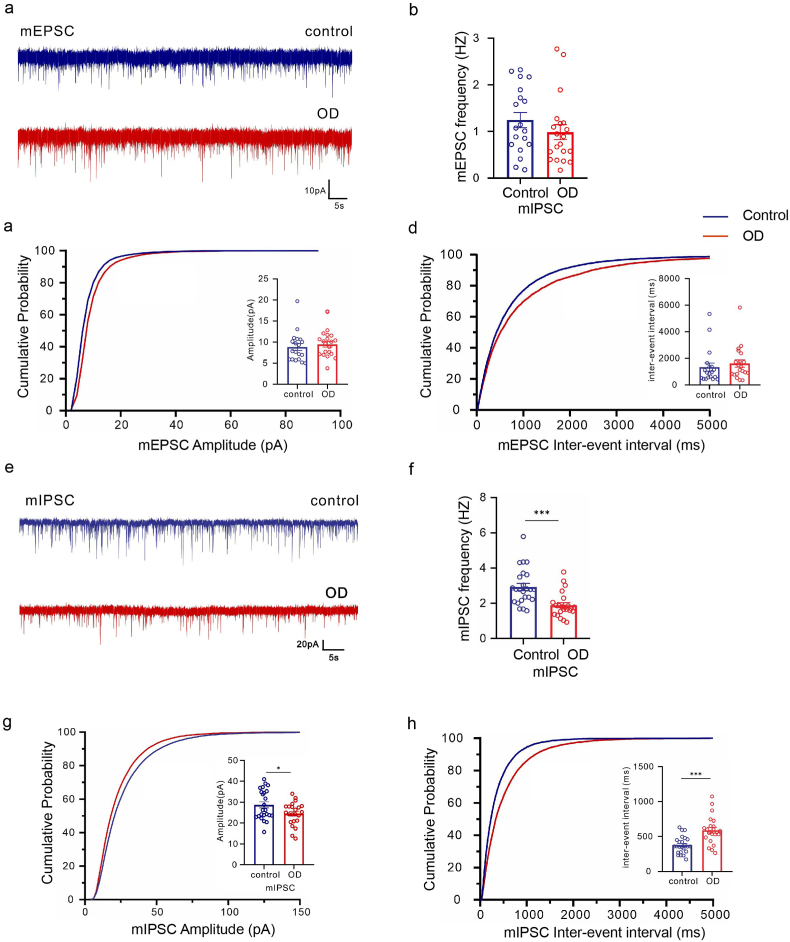
Inhibitory synaptic dysfunction in the mPFC pyramidal neurons of control and OD mice. (A) Representative mEPSC. (B) The frequency of mEPSC. Corresponding cumulative distributions and quantification of mEPSC (C)amplitudes and (D) frequencies. (n = 20 from 4 to 6 mice per group). (E) Representative mIPSC. (F)The frequency of mIPSC. (n = 20 from 4 to 6 mice per group). (G–H) Corresponding cumulative distributions and quantification of mEPSC (G)amplitudes and (H) frequencies (n = 20 from 4 to 6 mice per group). All data are presented as mean ± SEM; ∗, *p* < 0.05, ∗∗, *p* < 0.01, ∗∗∗, *p* < 0.001, by unpaired Student's t-test; Scale bar, 10 pA and 5s.

To further explore the functional influence of OD on synaptic transmission, the whole cell patch clamp technique was used to record mEPSC and mIPSC of pyramidal neurons in the mPFC, respectively. Notably, OD significantly reduces mIPSCs frequency by almost 50 %, while reducing the amplitude only by 15 % in mPFC pyramidal neurons [Fig. 4E–H], with no discernible impact on mEPSCs [Fig. 4A–D]. These findings demonstrated that OD induces a diminished inhibitory synaptic transmission in mPFC pyramidal neurons.

### 3.5 OD reduces the expression of NRG1 and ErbB4 in the mPFC

**Fig. 5:**
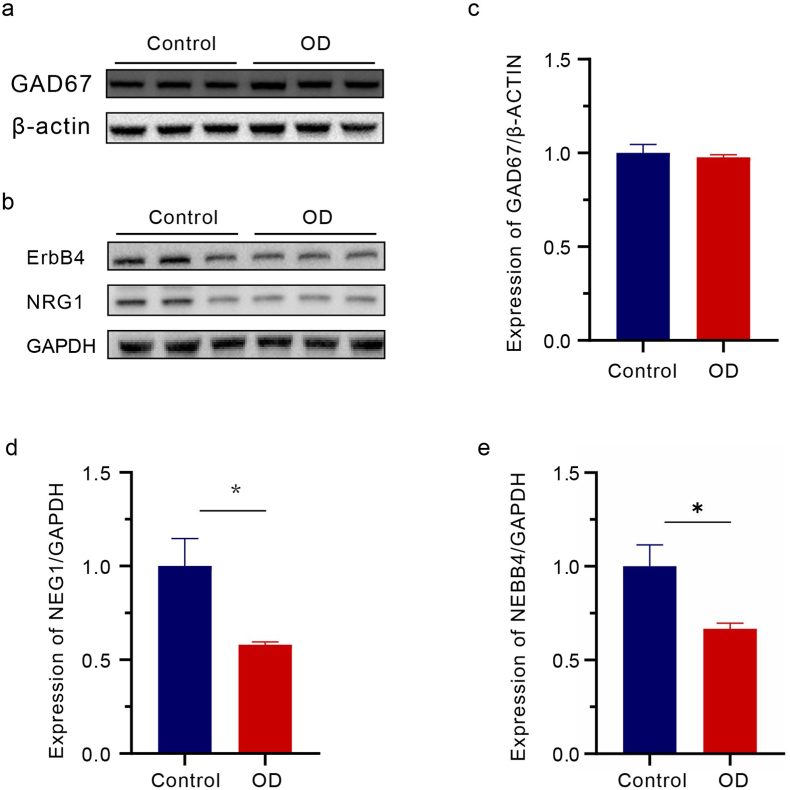
OD reduced NRG1 and ErbB4 expression in mPFC. (A–B) Representative western blots of GAD67, NRG1 and ErbB4 in the mPFC of mice exposed to OD for 14 days. (C–E) Quantitative data of the relative protein expression levels (n = 3). All data are presented as mean ± SEM; ∗, *p* < 0.05, ∗∗, *p* < 0.01, ∗∗∗, *p* < 0.001, by unpaired Student's t-test.

Considering the observed changes in inhibitory synaptic transmission in the mPFC and the predominance of GABAergic inhibitory neurons in the mPFC, the expression of proteins related to GABA synthesis (GAD67) and release (NRG1 and ErbB4) were assessed through Western blotting. The results show that NRG1 and ErbB4 exhibited a significant reduction [Fig. 5 B&D-E] after OD, while GAD67 showed no significant change when compared with the control group [Fig. 5 A&C]. These results suggested that altered GABA release may be the primary contributor to increased neuronal excitability in OD mice.

## 4 Discussion

Here, we found that OD, as a chronic systemic stressor, was the cause of anxiety in mice. This could be significantly alleviated by injecting mifepristone, a glucocorticoid receptor antagonist, further confirming that OD as a chronic stressor can lead to anxiety-like behavior in mice. In addition, our study reports for the first time that the mPFC is involved in the mechanism of OD-induced anxiety. OD reduced the amplitude and frequency of neuronal mIPSCs in the mPFC and ultimately increased the activity of mPFC neurons. Furthermore, OD decreased the expression of proteins associated with GABA release in the mPFC, whereas the expression of proteins related to GABA synthesis was not significantly different. In conclusion, these data suggest that the dysregulated GABAergic synaptic transmission system in the mPFC of OD rats results in E/I imbalance and may be a major neural mechanism for OD-induced anxiety.

People's occlusion changes throughout life. For example, in the natural physiological state, teeth erupt and attrition; in the pathological state, teeth suffer caries, loosening, and loss; or in the process of oral treatment, such as resin-filled restorations, fixed or removable prosthetic restorations, orthodontics, and surgical extractions, which change both the shape of the occlusal surfaces and the contacts of the teeth. Failure to restore occlusion in time can lead to several complex complications, one of which is anxiety [18,19]. However, the mechanism of OD-induced anxiety is complex, involving both oral science and neuroscience, and it is challenging to understand the mechanism and effectively treat patients. In this study, a mouse model of OD was constructed by gluing a metal tube to the right mandibular incisor of mice, which better simulated the clinical situation. Compared with other reported OD mouse construction methods [1,8,14,19], this method used metal materials, which made the tubes more stable on the teeth and less susceptible to wear. It also reduced the difficulty of the modeling operation, which greatly increased the success rate of modeling. In addition, the study showed that the body weight of the OD mice returned to normal after 5 days of modeling, suggesting that this modeling method did not have a lasting negative effect on the feeding activity, thus avoiding the possibility of false positives due to altered mobility of the mice and ultimately affecting the results of behavior tests.

Consistent with previously reported findings [1,8,9,20,21], OD mice exhibited anxiety-like behavior with decreased movement distance in the central region of the OFT, and reduced time and number in the open arms of the EPM. Moreover, OD activated the HPA axis and increased the serum CORT levels in mice. The HPA axis is a crucial stress-related central system that regulates organ metabolism and maintains homeostasis through a negative feedback pathway. CORT is a significant hormone in this process and is closely linked to mood. It is synthesized and secreted by the adrenal cortex and acts through negative feedback on GR receptors in the pituitary and various sites in the brain [22,23]. Therefore, it is deducible that GR may be a crucial therapeutic target for the abnormally activated HPA axis in OD-induced mood disorders. Mifepristone, a non-selective glucocorticoid receptor antagonist, has been shown to have favorable antidepressant and anxiolytic properties [24,25]. Therefore, we administered mifepristone daily to OD mice and observed a significant reduction in anxiety-related behaviors. These findings collectively suggest that chronic OD could lead to anxiety-like behaviors in mice by activating the HPA axis, inhibiting which could in turn alleviate the abnormal emotions in OD mice.

HPA axis is modulated by multiple emotion-related brain regions, with particular emphasis on the medial prefrontal cortex (mPFC) as a key regulatory hub for emotional processing. Our study specifically investigates the mPFC's role in this neuroendocrine circuitry. Existing evidence demonstrates that various stressors - including physical environmental challenges and psychosocial factors - can induce both structural and functional impairments in the mPFC. These neurobiological alterations have been consistently linked to the emergence of negative affective states [12,26,27]. In this study, immunofluorescence staining of c-FOS revealed that OD resulted in abnormally activated neurons in mPFC. Additionally, the whole-cell patch results demonstrated that OD could reduce inhibitory neurotransmission and E/I imbalance in vertebral neurons of mPFC. As GABA is the primary inhibitory neurotransmitter in the mPFC, it plays a vital role in maintaining the balance of E/I. Abnormalities in GABAergic neurons can result in various psychiatric disorders [28,29]. GABA is produced from the decarboxylation of glutamate, catalyzed by glutamate decarboxylases (GADs). Moreover, over 90 % of GABA synthesis in the brain is primarily dependent on GAD67. In the meanwhile, neuromodulin-1 (NRG1), a neurodevelopmental modulator, regulates inhibitory synapse formation, GABA release, and neurotransmission in vivo [30] by binding to its receptor ERBB4 at GABAergic terminals in the mPFC [[30], [31], [32]]. NRG1-ERBB4 signaling has been reported to maintain GABAergic activity in the amygdala and regulate anxiety-like behavior [33]. In the present study, the expression of NRG1 and ERBB4 was reduced in the mPFC of OD mice, while GAD67 remained unchanged. This indicates that reduced GABA release in mPFC pyramidal neurons and reduced inhibitory neurotransmission, leading to an E/I imbalance, ultimately contribute to anxiety-like behavior in mice.

In a word, this study provides compelling evidence indicating that the anomalous activation of the mPFC in mice with OD is attributable to a discernible reduction in GABA release, thereby precipitating anxiety-like behavior. However, further research is needed to confirm mechanism in-depth,such as using voltage clamp and HPLC to measure receptor kinetics or extracellular GABA levels, incorporating sEPSC/sIPSC recordings to investigate synaptic function under more physiological conditions, and identifying the brain regions that transmit oral information to the mPFC using viral tracing techniques. Besides, the specific role of distinct mPFC sub-regions (such as IL and PrL) and other emotions-related brain regions (like the hypothalamus, amygdala, and hippocampus) in OD-induced anxiety remain a critical area for exploration. Furthermore, this study is limited by the exclusive use of male mice. Although it was necessary to avoid confounding effects from estrous cycle-driven hormonal fluctuations in females, which are known to influence anxiety and synaptic function [34,35], potential sex differences in susceptibility to OD-induced anxiety and associated mPFC inhibitory synaptic alterations still need to be explored.

## 5 Conclusions

Our experiments have revealed the neural mechanism underlying OD-induced anxiety: we found that there was a decrease in GABA release and synaptic function in the mPFC of OD mice. It enhanced the excitability of mPFC, which resulted in anxiety-like behaviors in the mice. At the same time, the HPA axis was activated, but the use of appropriate inhibitors alleviated OD-induced anxiety. These findings offer new insights into the relationship between occlusion and mood and could assist in developing novel therapeutic strategies.

## CRediT authorship contribution statement

Juan Li: Data curation, Formal analysis, Methodology, Writing – original draft. Jiayao Zhang: Methodology, Visualizatio, Writing – original draft. Ming Xu: Data curation, Validation. Qi Zhang: Supervision, Writing – review and editing. Weicai Liu: Funding acquisition, Supervision, Writing – review and editing.

## Funding

This work was supported by the 10.13039/501100001809National Natural Science Foundation of China [grant numbers: 8227032547] and Natural Science Foundation of Shanghai [grant numbers: 20ZR1463000].

## Declaration of competing interest

The authors have no relevant financial or non-financial interests to disclose.

## Data Availability

Data will be made available on request.
